# Supplementary material for: Adaptive Compensatory Neurophysiological Biomarkers of Motor Recovery Post-Stroke: Electroencephalography and Transcranial Magnetic Stimulation Insights from the DEFINE Cohort Study
Source: Brain Sci. 2024 Dec 15;14(12):1257. doi: 10.3390/brainsci14121257 (PMC11674877; doi:10.3390/brainsci14121257)
Supplement: Supplementary file 1 [file brainsci-14-01257-s001.zip › brainsci-3311097-supplementary.pdf]

Supplementary Materials

Fugl-Meyer Assessment

The Fugl-Meyer Assessment (FMA) is a performance-based impairment index for patients with post-stroke hemiplegia. It is designed to independently assess various domains including motor skills, balance, sensation, and joint function in both upper and lower extremities. For this study, FMA was focused only on evaluating the motor function of the upper limb, with scores ranging from 0 (representing complete hemiplegia) to 66 points (2).

Hand grip and Pinching

Handgrip Strength Test (HST) and Pinch Strength Test (PST) evaluate muscle strength using a kg-strength scale measured by specific dynamometers. The HG test uses a hand grip dynamometer to measure the maximum force exerted during a five-finger squeeze. For the PT test, a pinch dynamometer is placed between the radial side of the index finger and thumb, and the participant is instructed to pinch as hard as possible. Both tests are performed with the participant seated, arms by the torso, elbows bent at 90 degrees, forearms in a neutral position, and wrists slightly extended. Each test is conducted three times on each hand, and the average strength is calculated (1,4,5).

Finger Tapping

The Finger Tapping (FTT) evaluates the speed of simple motor movements. In this test, which is conducted separately for each hand, the patient is instructed to tap a lever on a wooden board repeatedly with only their index finger for 30 seconds (6).

Nine-Hole Peg Test

The nine-hole (9HPT) peg test is a mechanical tool used to assess finger dexterity. During the test, a participant is required to use only one hand to sequentially pick up nine pegs from a container and insert them into the holes on a board in any order. After all pegs are placed, they must be removed one by one using the same hand. The duration recorded for the test is from when the first peg is picked up until the last peg is either returned to the container or set on the table (3).

References for the supplemental material

1. Boissy P, Bourbonnais D, Carlotti MM, Gravel D, Arsenault BA. Maximal grip force in chronic stroke subjects and its relationship to global upper extremity function. Clin Rehabil. 1999 Aug;13(4):354–62.
2. Gladstone DJ, Danells CJ, Black SE. The fugl-meyer assessment of motor recovery after stroke: a critical review of its measurement properties. Neurorehabil Neural Repair. 2002 Sep;16(3):232–40.
3. Jobbágy Á, Marik AR, Fazekas G. Quantification of the Upper Extremity Motor Functions of Stroke Patients Using a Smart Nine-Hole Peg Tester. J Healthc Eng. 2018;2018:7425858.
4. Mathiowetz V, Kashman N, Volland G, Weber K, Dowe M, Rogers S. Grip and pinch strength: normative data for adults. Arch Phys Med Rehabil. 1985 Feb;66(2):69–74.
5. Schmidt RT, Toews JV. Grip strength as measured by the Jamar dynamometer. Arch Phys Med Rehabil. 1970 Jun;51(6):321–7.
6. Shimoyama I, Ninchoji T, Uemura K. The finger-tapping test. A quantitative analysis. Arch Neurol. 1990 Jun;47(6):681–4.
